# Intermicrobial interaction: *Aspergillus fumigatus* siderophores protect against competition by *Pseudomonas aeruginosa*

**DOI:** 10.1371/journal.pone.0216085

**Published:** 2019-05-08

**Authors:** Gabriele Sass, Shajia R. Ansari, Anna-Maria Dietl, Eric Déziel, Hubertus Haas, David A. Stevens

**Affiliations:** 1 California Institute for Medical Research, San Jose, California, United States of America; 2 Division of Molecular Biology, Biocenter, Medical University of Innsbruck, Innsbruck, Austria; 3 INRS-Institut Armand-Frappier, Laval, Quebec, Canada; 4 Division of Infectious Diseases and Geographic Medicine, Department of Medicine, Stanford University School of Medicine, Stanford, California, United States of America; Leibniz-Institut fur Naturstoff-Forschung und Infektionsbiologie eV Hans-Knoll-Institut, GERMANY

## Abstract

*Pseudomonas aeruginosa* and *Aspergillus fumigatus* are pathogens frequently co-inhabiting immunocompromised patient airways, particularly in people with cystic fibrosis. Both microbes depend on the availability of iron, and compete for iron in their microenvironment. We showed previously that the *P*. *aeruginosa* siderophore pyoverdine is the main instrument in battling *A*. *fumigatus* biofilms, by iron chelation and denial of iron to the fungus. Here we show that *A*. *fumigatus* siderophores defend against anti-fungal *P*. *aeruginosa* effects. *P*. *aeruginosa* supernatants produced in the presence of wildtype *A*. *fumigatus* planktonic supernatants (Afsup) showed less activity against *A*. *fumigatus* biofilms than *P*. *aeruginosa* supernatants without Afsup, despite higher production of pyoverdine by *P*. *aeruginosa*. Supernatants of *A*. *fumigatus* cultures lacking the *sidA* gene (AfΔ*sidA*), unable to produce hydroxamate siderophores, were less capable of protecting *A*. *fumigatus* biofilms from *P*. *aeruginosa* supernatants and pyoverdine. AfΔ*sidA* biofilm was more sensitive towards inhibitory effects of pyoverdine, the iron chelator deferiprone (DFP), or amphothericin B than wildtype *A*. *fumigatus* biofilm. Supplementation of *sidA*-deficient *A*. *fumigatus* biofilm with *A*. *fumigatus* siderophores restored resistance to pyoverdine. The A. fumigatus siderophore production inhibitor celastrol sensitized wildtype *A*. *fumigatus* biofilms towards the anti-fungal activity of DFP. In conclusion, *A*. *fumigatus* hydroxamate siderophores play a pivotal role in *A*. *fumigatus* competition for iron against *P*. *aeruginosa*.

## Introduction

Ecosystems of pathogens have been described with regard to a multitude of diseases [1–3]. The bacterium *Pseudomonas aeruginosa* and the fungus *Aspergillus fumigatus* form such an ecosystem, e.g. when chronically colonizing the lungs of cystic fibrosis (CF) individuals [4–7]. Both pathogens have been associated with deterioration of lung function [4–17], and their combined presence in airways of CF patients seems to aggravate disease progression [18,19]. *P*. *aeruginosa* and *A*. *fumigatus* also are prominent opportunistic pathogens in immune-compromised patients, particularly in those with neutropenia [20,21].

Previous studies have focused on *A*. *fumigatus* inhibition caused by *P*. *aeruginosa* products such as pyocyanin (5-N-methyl-1-hydroxyphenazine) [22–25], 1-hydroxyphenazine [22,24,25], phenazine-1-carboxamide and phenazine-1-carboxylic acid [25]. We recently reported that the *P*. *aeruginosa* product pyoverdine is the major mediator of *P*. *aeruginosa* inhibitory function towards *A*. *fumigatus* biofilms [26]. Pyoverdine, the major siderophore of *P*. *aeruginosa* [27,28], strongly binds to iron, which is an essential co-factor for both *P*. *aeruginosa* and *A*. *fumigatus* [29–31]. Pyoverdine-bound iron is no longer available for *A*. *fumigatus*, starving *A*. *fumigatus* of iron, and resulting in fungistasis [26]. The question arose whether *A*. *fumigatus* could counteract *P*. *aeruginosa* inhibition. Here we provide evidence that *A*. *fumigatus* hydroxamate siderophores in times of iron shortage, created by a competing microbe, ensure availability of the essential co-factor iron exclusively to the fungus. Concomitantly, interference with *A*. *fumigatus* siderophore production renders the fungus more sensitive to anti-fungal effects of iron chelators, and possibly more sensitive even to effects of anti-fungal drugs not involved in iron chelation, like amphotericin B.

## Materials and methods

### Materials

Pyoverdine (PYOV), 3-hydroxy-1,2-dimethyl-4(1H)pyridine (deferiprone, DFP), celastrol, 2,3-bis[2-methoxy-4-nitro-5-sulfophenyl]-2H-tetrazolium-5-carboxanilide inner salt (XTT), and menadione were purchased from Sigma-Aldrich (St. Louis, MO). Amphotericin B (AmB) was derived from X-Gen Pharmaceuticals Inc. (Horseheads, NY). Chrome Azurol S (CAS) was purchased from MP Biomedicals (Solon, OH). Ferri- and desferri-triacetylfusarinine C (TAFC, DF-TAFC) were purified as described previously [32].

### Isolates

All isolates used in this study are summarized in Table 1.

**Table 1:** Isolates used in this study.

Organism	Isolate	Description	ATCC	Reference
*A*. *fumigatus*	10AF	Virulent patient isolate	90240	[33,34]
*A*. *fumigatus*	AF13073	Parental strain for AfΔ*sidA*	13073	
*A*. *fumigatus*	AfΔ*sidA*	l-ornithine-*N* ^5^-mono-oxygenase deficient *A*. *fumigatus* mutant strain		[35]
*A*. *fumigatus*	AF46645	Parental strain for AfΔ*sidC* and AfΔ*sidF*	46645	
*A*. *fumigatus*	AfΔ*sidC*	Deficient for the hydroxamate siderophores ferricrocin (FC) and hydroxy-FC (HFC)		[36]
*A*. *fumigatus*	AfΔ*sidF*	Deficient for the hydroxamate siderophores fusarinine C (FsC) and triacetylfusarinine C (TAFC).		[36]
*A*. *fumigatus*	AfS77	Derivate of ATCC 46645		[37]
*P*. *aeruginosa*	PA14	Parental strain for *pvdD-* and *pvdD-pchE-*		[38]
*P*. *aeruginosa*	*pvdD-*	Pyoverdine deficient mutant		[39]
*P*. *aeruginosa*	*pvdD-pchE-*	Pyoverdine/pyochelin deficient mutant		[26]

### The work flow for the following procedures is summarized in S1 Fig.*A*. *fumigatus* supernatant production

*A*. *fumigatus* conidia were inoculated into RPMI 1640 medium (RPMI, Lonza, Walkersville, MD) at 2.5x10^4^ conidia/ml. *A*. *fumigatus* suspensions were incubated at 37°C for 48h (S1 Fig). *A*. *fumigatus* supernatants (Afsup) were filtered (0.22 μm) for sterility after the growth period.

### *Pseudomonas* supernatant production and pyoverdine measurement

PA14 supernatants were prepared as detailed previously [40]. Briefly, *P*. *aeruginosa* [5 x 10^7^ cells/ml] was inoculated into RPMI 1640 medium, or mixtures of RPMI and Afsup, and incubated at 37°C for 24h. Bacterial growth was measured at 600 nm with a spectrophotometer (Genesys 20, Thermo Fisher Scientific Inc., Waltham, MA). Bacterial cultures were centrifuged at 200 x *g* for 30 min at room temperature, and filtered (0.22 μm). Pyoverdine production in the supernatant was measured as described previously [41] at 405 nm. Pyoverdine measurements were normalized to bacterial growth using the formula: Relative PYOV expression = OD405 / OD600. At the concentrations used in this study, pyoverdine, a colored substance, did not interfere with the colorimetric XTT assay used for determination of fungal metabolism. PYOV concentrations in undiluted *P*. *aeruginosa* supernatants are about 30 μM. Pyoverdine concentrations in sputum have been shown to be between 0.3 and 51 μM [42].

### Assay for the measurement of metabolism of *A*. *fumigatus* forming (BCAM assay, Bioassay-Conidia-Agar-Metabolic) or preformed (BHAM assay, Bioassay-Hyphae-Agar-Metabolic) biofilms

BCAM and BHAM assays were performed as described previously [26]. In these assays, *A*. *fumigatus* grows out into biofilms covering the agar surface. Briefly, RPMI agar containing 2.5x10^4^ to 10^5^ *A*. *fumigatus* conidia/ml agar (as specified for different experiments in the Results section) was distributed into sterile flat-bottom 96 well cell culture plates (COSTAR, Corning, NY) at 100 μl/well. Upon agar solidification, wells were either incubated at 37°C for 24 hours before loading (= BHAM assays), or immediately loaded with 100 μl of test substances (= BCAM assays). Control wells on each test plate contained 100 μl of RPMI 1640 medium, allowing the conversion of test results to % of the RPMI control (= 100%). Loaded plates were incubated at 37°C for 24 hours. Fungal metabolism was determined by XTT metabolic assay at 490 nm [40,43]. Menadione (vitamin K3) was used as an ingredient in the XTT metabolic assay, boosting the reduction of tetrazolium salts to formazans. XTT assays were evaluated using a plate reader (Opsys MR, DYNEX Technologies, Chantilly, VA). Although XTT is a measure of metabolic activity of cells, previous studies of *A*. *fumigatus* have indicated that XTT results are linear with mass, and equated XTT result with dry weight [44–46].

### *Aspergillus* growth assays

AfΔ*sidA* (10^4^conidia) was point-inoculated on 2 ml solid minimal medium [47] in the presence of 50–600 μl PA14 wildtype or PA14 PaΔ*pvdD* bacterial supernatant with or without supplementation of FeSO_4_ [1 μM]. Radial fungal growth was scored after incubation of the plates for 48 hours at 37°C.

### Chrome azurol S (CAS) assay

For measurement of siderophore production 10x CAS assay reagent was prepared as described previously [48]. One part 10x CAS reagent was combined with 9 parts Afsups in RPMI, and incubated at 37°C for 24 hours. Mixtures were measured using the plate reader, and compared to RPMI not containing CAS reagent or RPMI/1x CAS reagent as reference points.

### Statistical analysis

Results were analyzed using Student’s *t* test, if two groups were compared, and by 1-way ANOVA, combined with a Tukey’s post-test for multiple comparisons. Data reported as percentages of the control value were compared after arcsin transformation of the proportions [26]. All data in this study are expressed as a mean ± SD.The number of replicates in each assay is four or higher. Assays were repeated at least twice, and a representative experiment is shown. Supporting information on data sets used in this study is provided in S1 Table.

## Results

### *A*. *fumigatus* supernatants induce pyoverdine production by *P*. *aeruginosa*

**Fig 1:**
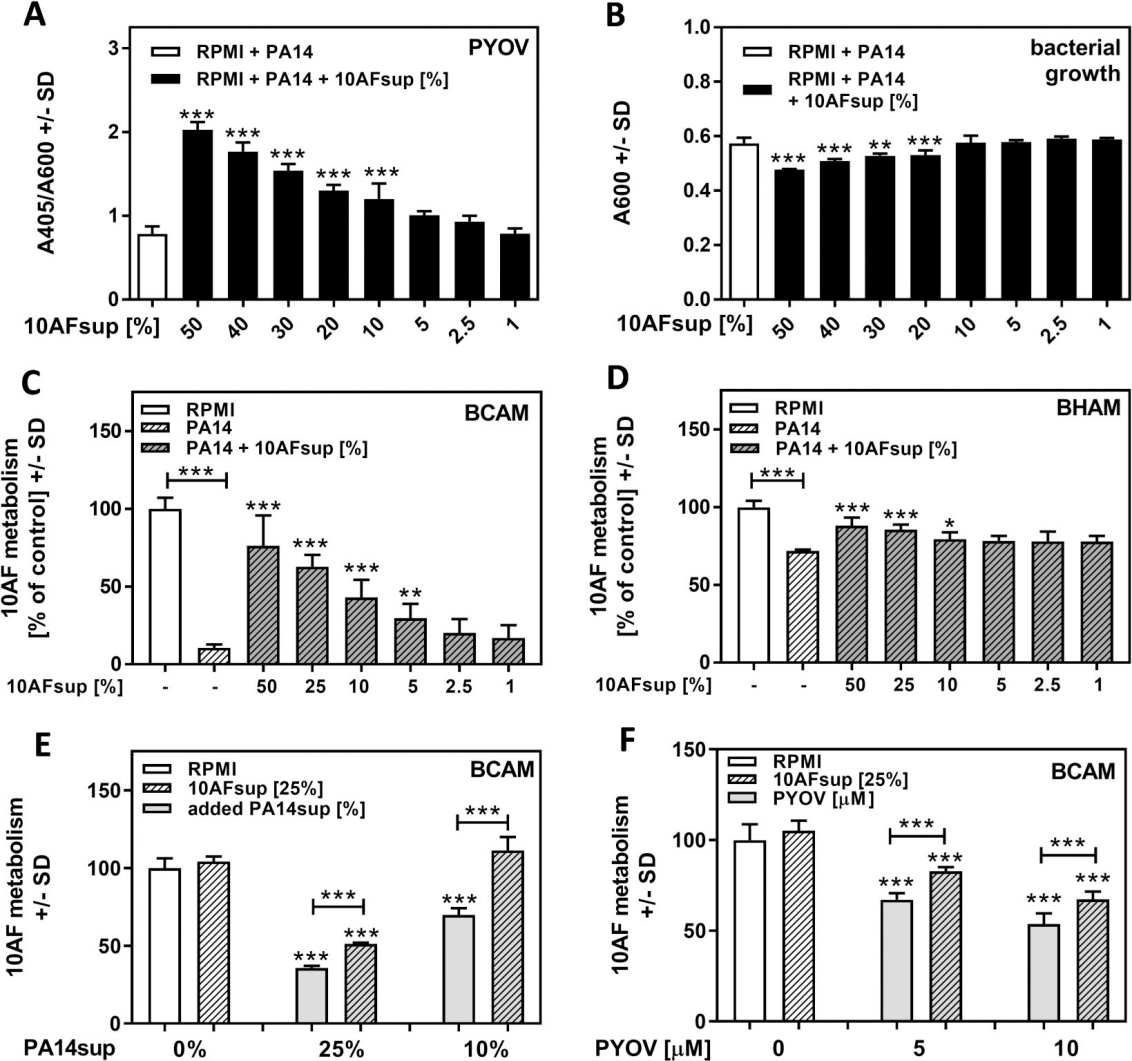
*A*. *fumigatus* supernatant effects on *P*. *aeruginosa growth and pyoverdine production*. Planktonic 10AF supernatant (10AFsup) was diluted in RPMI from 50% to 0% 10AFsup, and incubated with *P*. *aeruginosa* cells (5x10^7^/ml) at 37°C for 24h. Relative pyoverdine (PYOV) concentrations (A) were calculated using the quotient A405 (PYOV)/A600 (bacterial growth: B). Supernatants shown in A and B, as well as PA14 supernatant not containing 10Afsup, were compared with respect to their activities on 10AF forming biofilm (C: BCAM) or preformed biofilm (D: BHAM) metabolism. E: 10AF forming biofilm was incubated with 10 or 25% PA14 wildtype supernatant with or without the addition of 25% 10AFsup for 24 hours. Effects on 10AF forming biofilm metabolism were evaluated by XTT assay. F: 10AF forming biofilm was incubated with 5 or 10 μM pyoverdine (PYOV) with or without the addition of 25% 10AFsup for 24 hours. Effects on 10AF forming biofilm metabolism were evaluated by XTT assay. Statistics for A and B: 1way ANOVA, RPMI (white bar) vs. all groups containing Afsup (black bars). Statistics for C and D: 1way ANOVA, PA14 supernatant (white striped bar) vs. PA14 supernatant containing 10AFsup (grey striped bars). Other comparisons by t-Test as indicated by the ends of the brackets. Statistics for E and F: t-Test, RPMI (white bar) vs. all other bars. Other comparisons as indicated by the ends of the brackets. One, two or three asterisks = p ≤ 0.05, p ≤ 0.01 or p ≤ 0.001, respectively.

Fungal supernatants (Afsup), produced by planktonic growth of *A*. *fumigatus* strain 10AF in RPMI (experimental setup described in S1 Fig), induced pyoverdine production by *P*. *aeruginosa* in a dose-dependent manner, with 10% Afsup still significantly inducing pyoverdine production. As pyoverdine is induced in response to iron shortage, increased pyoverdine production here suggests sequestration of iron from the growth medium by Afsup (Fig 1A).

Concentrations of Afsup higher than 10% interfered with bacterial growth in a concentration-dependent manner (Fig 1B). As iron is a major co-factor for microbial growth, the reason for inhibitory effects of Afsup on *P*. *aeruginosa* growth might be a reaction to iron denial. Although gliotoxin has been suggested as an anti-microbial factor [49], in our hands supernatants produced by an *A*. *fumigatus* mutant unable to produce gliotoxin [50] affected *P*. *aeruginosa* growth to a similar degree as supernatants produced by its parent (S2 Fig). Distilled water (25%), instead of Afsup (25%) during *P*. *aeruginosa* supernatant preparation did not result in interference with *P*. *aeruginosa* effects on *A*. *fumigatus* biofilm metabolism, indicating that *P*. *aeruginosa* supernatant dilution by Afsups was not the reason for the protective effects of Afsups (S3 Fig).

In order to verify that Afsup indeed induced production of pyoverdine, we used a PA14 mutant not able to produce pyoverdine (PaΔ*pvdD*) [39]. With or without the presence of Afsup, PaΔ*pvdD* supernatant did not absorb at 405 nm, confirming that Afsup did not induce production of an unknown *P*. *aeruginosa* product detectable at 405 nm (S4A Fig).

### Afsup protects *A*. *fumigatus* forming biofilm from *P*. *aeruginosa* anti-fungal activity and pure pyoverdine

Pyoverdine has detrimental effects on Af biofilm metabolism [26]. Surprisingly, although containing high concentrations of pyoverdine (Fig 1A), *P*. *aeruginosa* supernatants produced in the presence of Afsup were less inhibitory for forming (Fig 1C) or preformed (Fig 1D) *A*. *fumigatus* biofilms than *P*. *aeruginosa* supernatants produced without Afsups,whereas Afsups up to 50% did not affect *A*. *fumigatus* biofilms when administered alone. Similarly, when *P*. *aeruginosa* supernatants and Afsups were prepared separately, their combination was less inhibitory to *A*. *fumigatus* biofilms than *P*. *aeruginosa* supernatant alone (Fig 1E).

Presumably owing to their lack of pyoverdine production, supernatants of PaΔ*pvdD* were less inhibitory to 10AF biofilms than PA14 supernatants. The presence of Afsup further decreased the inhibitory activity of PaΔ*pvdD* supernatants to RPMI control levels (S4B Fig). Protective Afsup effects were also observed when Afsups were combined with pure pyoverdine (Fig 1F).

Taken together, these data indicate that despite its ability to induce pyoverdine production by *P*. *aeruginosa*, Afsup protects *A*. *fumigatus* biofilms.

### Stability of protective Af supernatant effects

**Fig 2:**
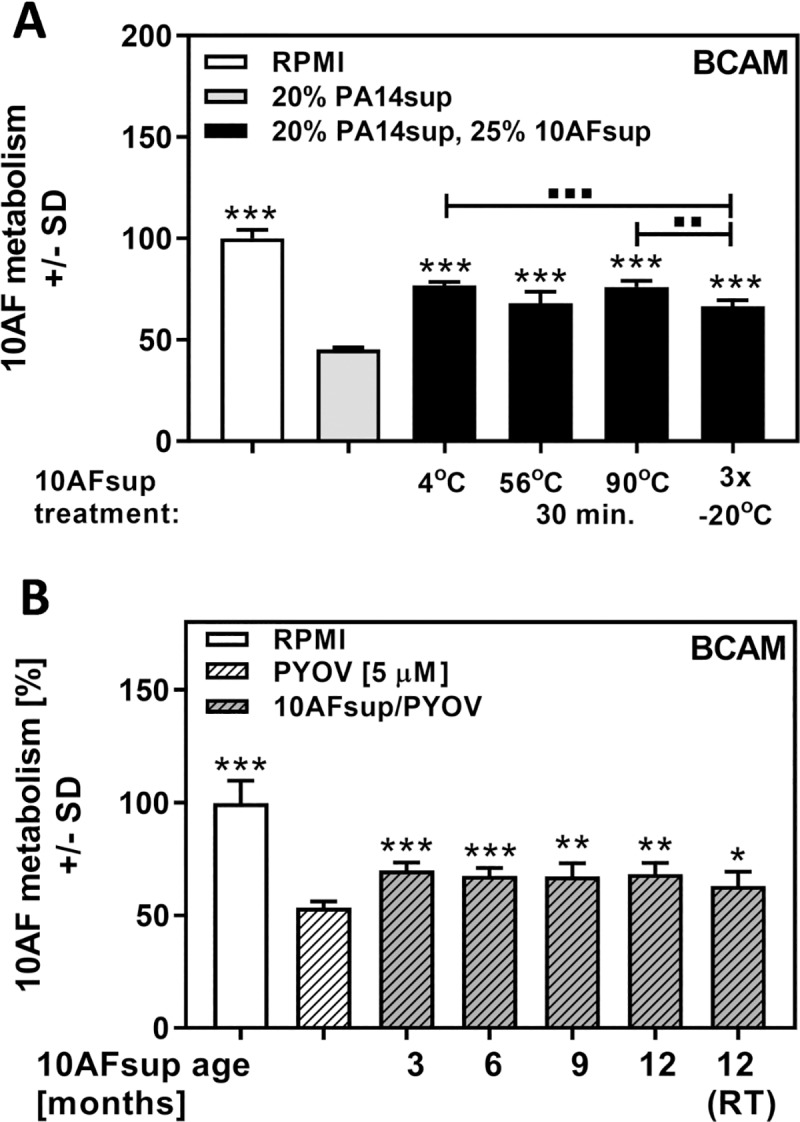
Stability of Afsup. A: Mixtures (25%) of freshly prepared 10AFsup in RPMI were kept at 4°C, or heated to 56°C or 90°C for 30 minutes, or subjected to 3 freeze-thaw cycles. Treated 10AFsups (25% in RPMI), were combined with 20% *P*. *aeruginosa* supernatants, and tested for effects on *A*. *fumigatus* forming biofilm metabolism. B: 10AFsup was stored at 4°C for up to 12 months, and tested for protective activity against 5 μM pyoverdine (PYOV) every 3 months. A portion of the 10AFsup was stored at room temperature (RT), and tested after 12 months of storage. Protective activity was tested using a BCAM assay. Statistics for A: t-Test, PA14 supernatant (grey bar) vs. all other bars. Other comparisons as indicated by the ends of the brackets. * indicate significant increases, ▪ indicate significant decreases. Statistics for B: t-Test, pyoverdine (white striped bar) vs. all other bars. One, two or three asterisks or squares = p ≤ 0.05, p ≤ 0.01 or p ≤ 0.001, respectively.

In order to determine the reason for protection of A. fumigatus biofilms by Afsup, we first tested stability of 10AFsup to heat, and long-term storage. 10AFsup was heated to 56°C or 90°C for 30 minutes, or subjected to three freeze-thaw cycles. Treated or untreated 10AFsups were diluted to 25%, combined with *P*. *aeruginosa* supernatants, and tested for effects on *A*. *fumigatus* biofilm metabolism. Our results show that heat does not destroy the protective compound in Afsup (Fig 2A). Repeated freeze-thaw cycles diminished, but did not abolish protection (Fig 2A). We also kept 10AFsup at 4°C for 12 months, and measured protection from pyoverdine every 3 months. The protective potential of Afsup was almost constant over the 12 months period (Fig 2B). When 10AFsup was kept at room temperature for 12 months, protection was marginally lower, but still significant (Fig 2B). Taken together, our data suggest that the protective compound in 10AFsup is stable.

### *A*. *fumigatus* siderophores protect *A*. *fumigatus* biofilm from *P*. *aeruginosa* anti-fungal activity

**Fig 3:**
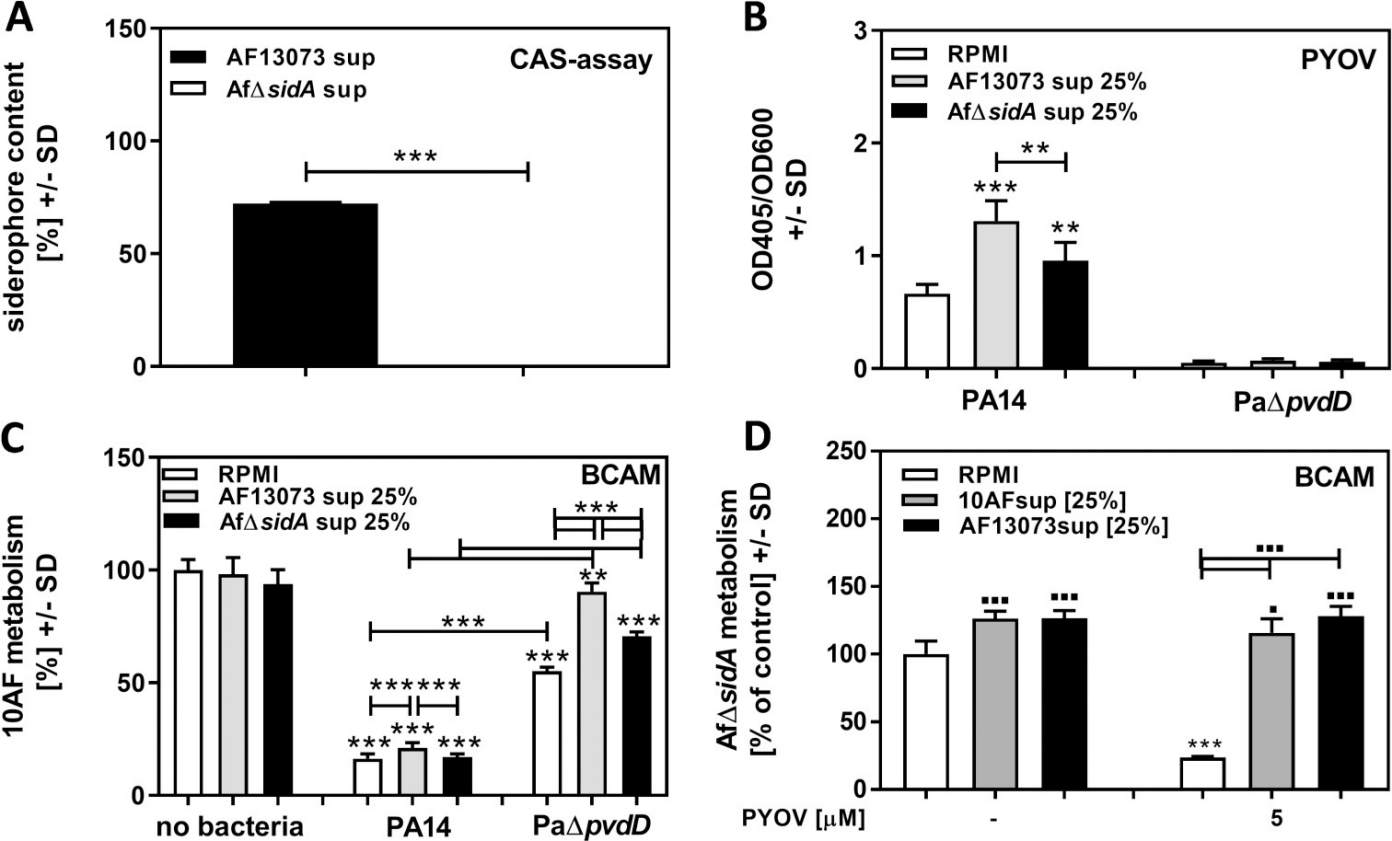
*A*. *fumigatus* siderophores protect *A*. *fumigatus* biofilm from *P*. *aeruginosa* anti-fungal activity. A: Planktonic supernatants produced by an *A*. *fumigatus* mutant lacking hydroxamate siderophore production (AfΔ*sidA*) or its parental strain (AF13073) were subjected to siderophore production measurement by CAS assay. B: RPMI, or 25% AfΔ*sidA* or AF13073 supernatant in RPMI, were inoculated with PA14 wildtype or the PA14 mutant PaΔ*pvdD* [5x10^7^ cells/ml], and incubated at 37°C for 24h. Pyoverdine production was measured. C: Supernatants obtained in B (middle and right sets of 3 bars), as well as 25% AfΔ*sidA* or AF13073 supernatants in RPMI (left 3 bars) were tested for activity against *A*. *fumigatus* biofilm formation. D: AfΔ*sidA* forming biofilm was incubated with 5 μM pyoverdine (PYOV) with or without the addition of 25% 10AFsup or AF13073sup for 24 hours. Effects on forming biofilm metabolism were evaluated by XTT assay. Statistics: t-Test. Comparisons without brackets: B: RPMI vs. *A*. *fumigatus* supernatants for each bacterial strain. C: RPMI vs. all other bars. D: RPMI (leftmost white bar) vs. all other bars. Other comparisons as indicated by the ends of the brackets. * indicate significant decreases, ▪ indicate significant increases. One, two, or three asterisks or squares = p ≤ 0.05, or p ≤ 0.01, or p ≤ 0.001, respectively.

Knowing that iron is a crucial factor for *A*. *fumigatus* biofilm, that pyoverdine inhibitory activity is owing to withholding iron from the fungus, and that the protective compound in Afsup is stable (Fig 2), we investigated the hypothesis that the protective compound might be an *A*. *fumigatus* siderophore. We produced supernatant of an *A*. *fumigatus* mutant lacking *sidA*, a gene crucial for the production of all four hydroxamate siderophores (AfΔ*sidAs*up), and compared to wildtype Afsup, produced by the AfΔ*sidA* parent AF13073 (AF13073sup). A CAS assay confirmed the lack of siderophores in AfΔ*sidA*sup (Fig 3A). Dilutions (25%) of AF13073sup and AfΔ*sidA*sup were incubated with PA14 or PaΔ*pvdD*. AF13073sup stimulated pyoverdine production by PA14 significantly more than AfΔ*sidA* sup (Fig 3B). AfΔ*sidA*sup also showed less protection for *A*. *fumigatus* biofilm against *P*. *aeruginosa* anti-fungal activity than AF13073sup (Fig 3C). When siderophore-deficient fungus was treated with pyoverdine, significant damage was induced (Fig 3D), whereas Afsup derived from either 10AF or AF13073 wildtype strains protected AfΔ*sidA* from pyoverdine-induced damage (Fig 3D). It has to be noted that neither the absence of pyoverdine nor the presence of Afsup from siderophore-deficient fungus prevented *P*. *aeruginosa* anti-fungal activity completely.

In comparison to wildtype *A*. *fumigatus*, AfΔ*sidA* has a growth disadvantage due to missing Fe^3+^ uptake, which requires siderophores. 10AF or AF13073 wildtype supernatants, containing siderophores and iron, partially compensated AfΔ*sidA* disadvantages, as indicated by higher XTT values for AfΔ*sidA* in the presence of Afsups (Fig 3D). In conclusion, *A*. *fumigatus* siderophores are able to protect *A*. *fumigatus* biofilms against *P*. *aeruginosa* anti-fungal activity. Fig 3C also shows that AfΔ*sidA* sup was able to provide protection for *A*. *fumigatus* biofilm from PaΔ*pvdD* supernatant, whereas there was no protection against PA14 wildtype sup by either Afsup. This finding indicates that *A*. *fumigatus* hydroxamate siderophores are crucial for protection from detrimental pyoverdine effects, but that Afsup seems to contain other compounds which are able to protect *A*. *fumigatus* biofilm when the Pasup challenge lacks the powerful inhibitor pyoverdine.

### AfΔ*sidA* is more sensitive to *P*. *aeruginosa* anti-fungal activity and pyoverdine than its wildtype

**Fig 4:**
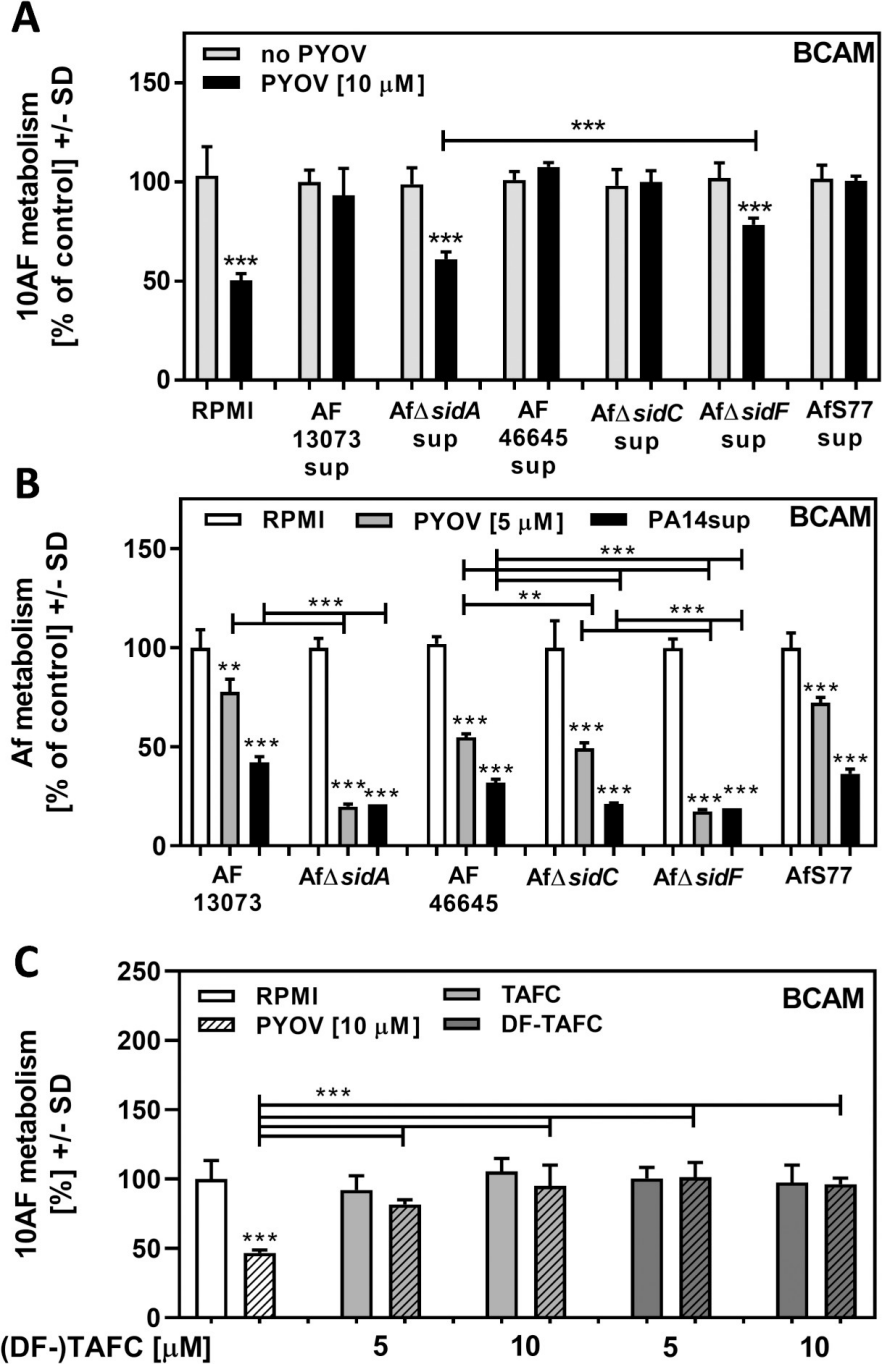
AfΔ*sidA* is more sensitive towards PA14 or pure pyoverdine than its wildtype. A: Mixtures (25%) of freshly prepared AF13073, AfΔ*sidA*, AF46645, AfΔ*sidC*, AfΔ*sidF*, or AfS77 supernatants were combined with pyoverdine [10 μM], and tested for effects on 10AF forming biofilm metabolism. Fungal metabolism was measured by XTT assay. Measurements for controls (no pyoverdine) in each group were regarded as 100%. Statistics: t-Test, for each group: no pyoverdine (grey bar) vs. pyoverdine (black bar). Other comparison as indicated by the ends of the bracket. B: AF13073, AfΔ*sidA*, AF46645, AfΔ*sidC*, AfΔ*sidF* or AfS77 BCAM assays were incubated with either RPMI, PA14 supernatant, or 5 μM pyoverdine. Fungal metabolism was measured by XTT assay. For each fungus RPMI control measurements were regarded as 100%. Statistics: t-Test, comparison: RPMI (white bars) vs. PA14 supernatant (grey bars), or pyoverdine (black bars) for each fungus. Other comparisons as indicated by the ends of the brackets. C: A 10AF BCAM assay was incubated with either RPMI, pyoverdine [10 μM], TAFC [5 or 10 μM], DF-TAFC [5 or 10 μM], or combinations of pyoverdine and TAFC or DF-TAFC. Fungal metabolism was measured by XTT assay. RPMI control measurements were regarded as 100%. Statistics: t-Test, comparison: RPMI (white bar) vs. all other bars. Other comparisons as indicated by the ends of the brackets. One, two or three asterisks = p ≤ 0.05, p ≤ 0.01 or p ≤ 0.001, respectively.

AfΔ*sidA-*derived supernatants were significantly less protective against pyoverdine than wildtype supernatants (Fig 4A). AfΔ*sidA* is lacking the intracellular hydroxamate siderophores ferricrocin (FC) and hydroxy-ferricrocin (HFC), as well as the extracellular hydroxamate siderophores fusarinin C (FsC) and triacetylfusarinine C (TAFC). Using *A*. *fumigatus* mutants with specific mutations in intracellular (AfΔ*sidC*), or extracellular hydroxamate siderophores (AfΔ*sidF*), we found that a lack of extracellular siderophores significantly interfered with protection from pyoverdine by *A*. *fumigatus* supernatants (Fig 4A). Protective effects of AfΔ*sidF* sup were significantly higher than protective effects of AfΔ*sidA* sup (Fig 4A), indicating that there might be some contribution to protection by other molecules missing in AfΔ*sidA* sup. Fig 4A also shows that supernatants, derived from three different *A*. *fumigatus* wildtypes (AF13073, AF46645, AfS77) protected forming biofilm of a fourth *A*. *fumigatus* wildtype (10AF), indicating that protection is not strain specific.

PA14 supernatants, prepared in RPMI, as well as pure pyoverdine, were significantly more inhibitory during the formation of *A*. *fumigatus* biofilms derived from AfΔ*sid*A conidia than they were for biofilms derived from AF13073 conidia (Fig 4B). *A*. *fumigatus* mutants lacking either intracellular (AfΔ*sidC*), or extracellular hydroxamate siderophores (AfΔ*sidF*) showed increased sensitivity to PA14 supernatants or pure pyoverdine, compared to their wildtype AF46645 (Fig 4B). The loss of extracellular hydroxamate siderophores was more important for sensitivity than the loss of intracellular hydroxamate siderophores (Fig 4B). Using pure TAFC, or desferri-TAFC (DF-TAFC) we found complete protection from pyoverdine anti-fungal activity (Fig 4C), confirming the importance for *A*. *fumigatus* siderophores for protection from *P*. *aeruginosa* anti-fungal activity.

As observed in Fig 2, the protective compound in Afsup was stable to prolonged heat treatment (90°C, 30 min.). After being subject to the same treatment pure TAFC and DF-TAFC still significantly protected from pyoverdine toxicity (S5 Fig), further supporting the assumption that *A*. *fumigatus* siderophores are the protective compound in Afsup. It was also noted that pyoverdine was heat stable (S5 Fig).

### The absence of A. fumigatus hydroxamate siderophores might have therapeutic relevance

**Fig 5:**
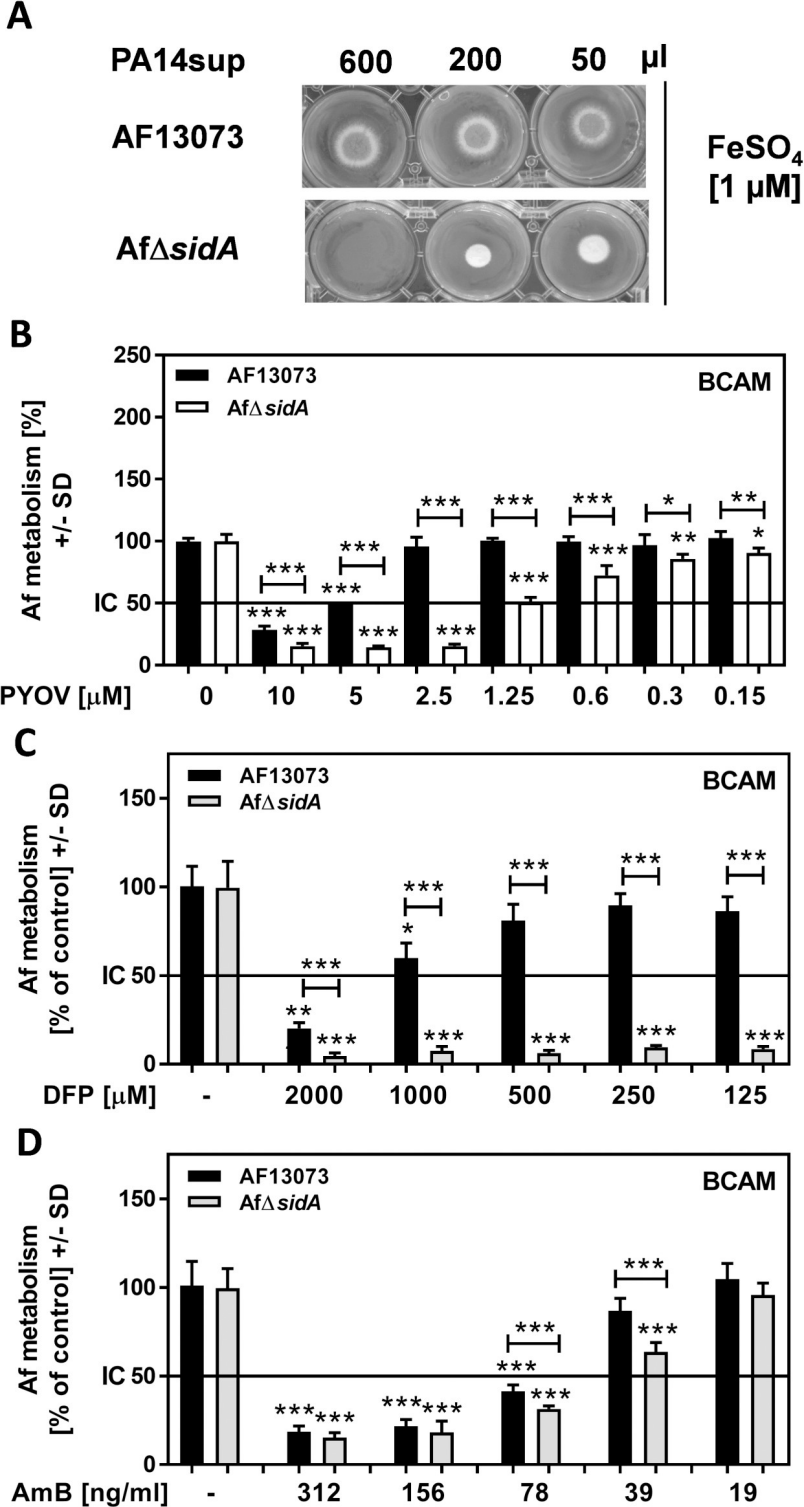
Absence of hydroxamate siderophores sensitizes *A*. *fumigatus*. A: RPMI was inoculated with PA14 [5x10^7^ cells/ml], incubated for 24 hours, and the culture supernatant was sterile filtered. Growth of point inoculated AF13073 or AfΔ*sidA* (10^4^conidia) on 3 ml solid minimal medium in the presence of 1 μM FeSO_4_ plus 50–600 μl of the sterile filtered supernatants was compared after incubation for 48 h at 37°C. B: AfΔ*sidA* (white bars) or AF13073 (black bars) BCAM assays were incubated with either RPMI or different concentrations of pyoverdine. Fungal metabolism was measured by XTT assay. Statistics: t-Test. For each fungus RPMI controls were regarded as 100%. RPMI controls for each fungus vs. all pyoverdine concentration. Other comparisons as indicated by the ends of the brackets. C: Wildtype (AF13073) or AfΔ*sidA* forming biofilms were incubated with DFP [0.125–2 mM] at 37°C for 24 hour. Fungal metabolism was measured by XTT assay. Statistics: t-Test, RPMI vs. all other bars of the same group. Other comparisons as indicated by the ends of the brackets. D: Wildtype (AF13073) or AfΔsidA forming biofilms were incubated with AmB [19–312 ng/ml] at 37°C for 24 hour. Fungal metabolism was measured by XTT assay. Statistics: t-Test, RPMI vs. all other bars of the same group. Other comparisons as indicated by the ends of the brackets. One, two or three asterisks = p ≤ 0.05, p ≤ 0.01 or p ≤ 0.001, respectively.

Compared to wildtype *A*. *fumigatus* (AF13073), AfΔ*sid*A growth on plate was more affected with the highest concentration (600 μl) of PA14 supernatant blocking growth (Fig 5A). Likewise, the IC50 of pyoverdine for AF13073-derived forming biofilm was about 4 times higher than the IC50 for AfΔ*sid*A-derived forming biofilm (Fig 5B). Recently, the iron chelator deferiprone (DFP), which similar to pyoverdine, exerts anti-fungal activity by denying iron from *A*. *fumigatus* biofilms, has been proposed to be useful in anti-fungal therapy [51,52]. We tested effects of DFP on AfΔ*sidA*, or its wildtype, and found significantly higher sensitivity of *A*. *fumigatus* biofilms to DFP when siderophore production was missing (Fig 5C). Genetic inhibition of siderophore production also increased anti-fungal effects of amphotericin B (AmB), an anti-fungal agent used against serious fungal infections, not only by *Aspergillus*, but also by other fungi [53], on *A*. *fumigatus* forming biofilm metabolism (Fig 5D).

**Fig 6:**
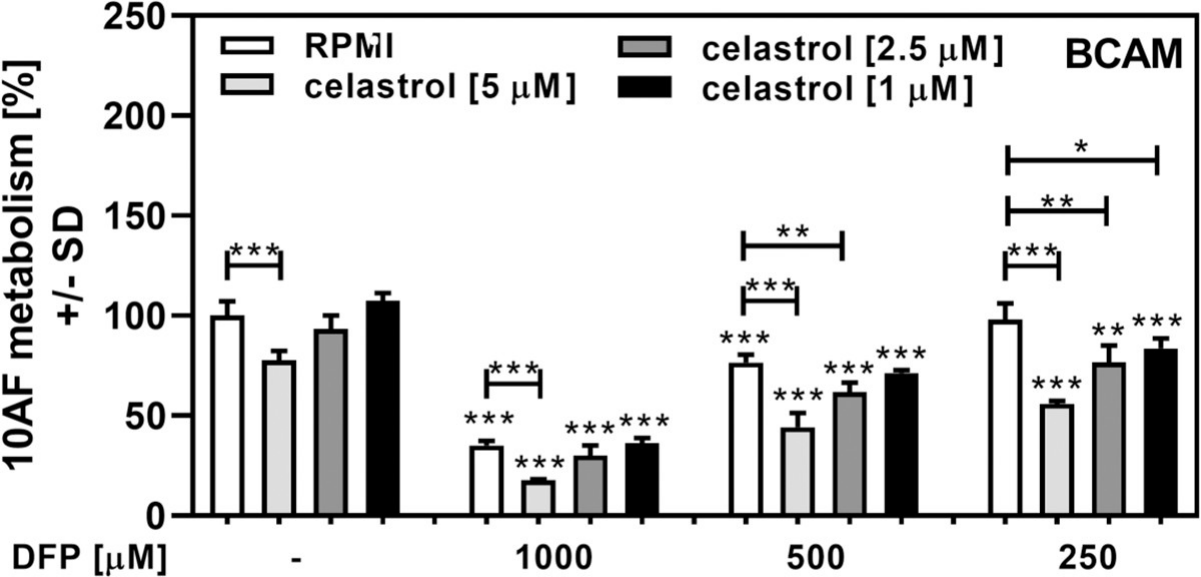
Celastrol sensitizes *A*. *fumigatus* for anti-fungal activity of DFP. Forming wildtype *A*. *fumigatus* biofilm (10AF) was incubated with 1, 2.5, or 5 μM celastrol, 0.25–1 mM of DFP, or combinations of these two substances at 37°C for 24 hour. Fungal metabolism was measured by XTT assay. Statistics: t-Test. Bars without DFP (leftmost group) vs. all other bars with the same celastrol concentration. Other comparisons as indicated by the ends of the brackets. One, two or three asterisks = p ≤ 0.05, p ≤ 0.01 or p ≤ 0.001, respectively.

As a pharmacological complementation of our data obtained using AfΔ*sidA*, we investigated effects of the SidA-biosynthesis inhibitor celastrol [54]. Celastrol showed anti-fungal activity when used alone at concentrations above 5 μM (Fig 6). When combined with DFP, celastrol significant enhanced anti-fungal effects by DFP (Fig 6).

## Discussion

Fungal and bacterial biofilms e.g. frequently found co-inhabiting lungs of persons suffering from cystic fibrosis, represent a potentially severe pathogenicity factor. The present study mainly focuses on events during formation of *A*. *fumigatus* biofilm. In previous studies [40] and in studies by many others, it has been shown that biofilm formation by *A*. *fumigatus* is substantial within the first 16 hours of incubation. We have also performed many of the studies described in the present communication against fully formed *A*. *fumigatus* biofilms that develop over the subsequent 24 hours of incubation, and found the same phenomena, although to a lesser degree than in the earlier phase of *A*. *fumigatus* biofilm formation, as illustrated in Fig 1C vs. 1D. This may suggest that iron is more important for the initial development of *A*. *fumigatus* biofilms.

The human body contains free iron levels of 10^−24^ M [55]. Free iron levels are decreased during infections due to increased levels of ferritin and the release of lactoferrin from neutrophils [56]. In the lungs of cystic fibrosis patients, *P*. *aeruginosa* and *A*. *fumigatus*, which both are crucially dependent on the availability of free iron for metabolism and growth, aggravate disease pathology [4–7]. Under low iron conditions, these organisms are forced to compete for resources in the same environment [29,30].

**Fig 7:**
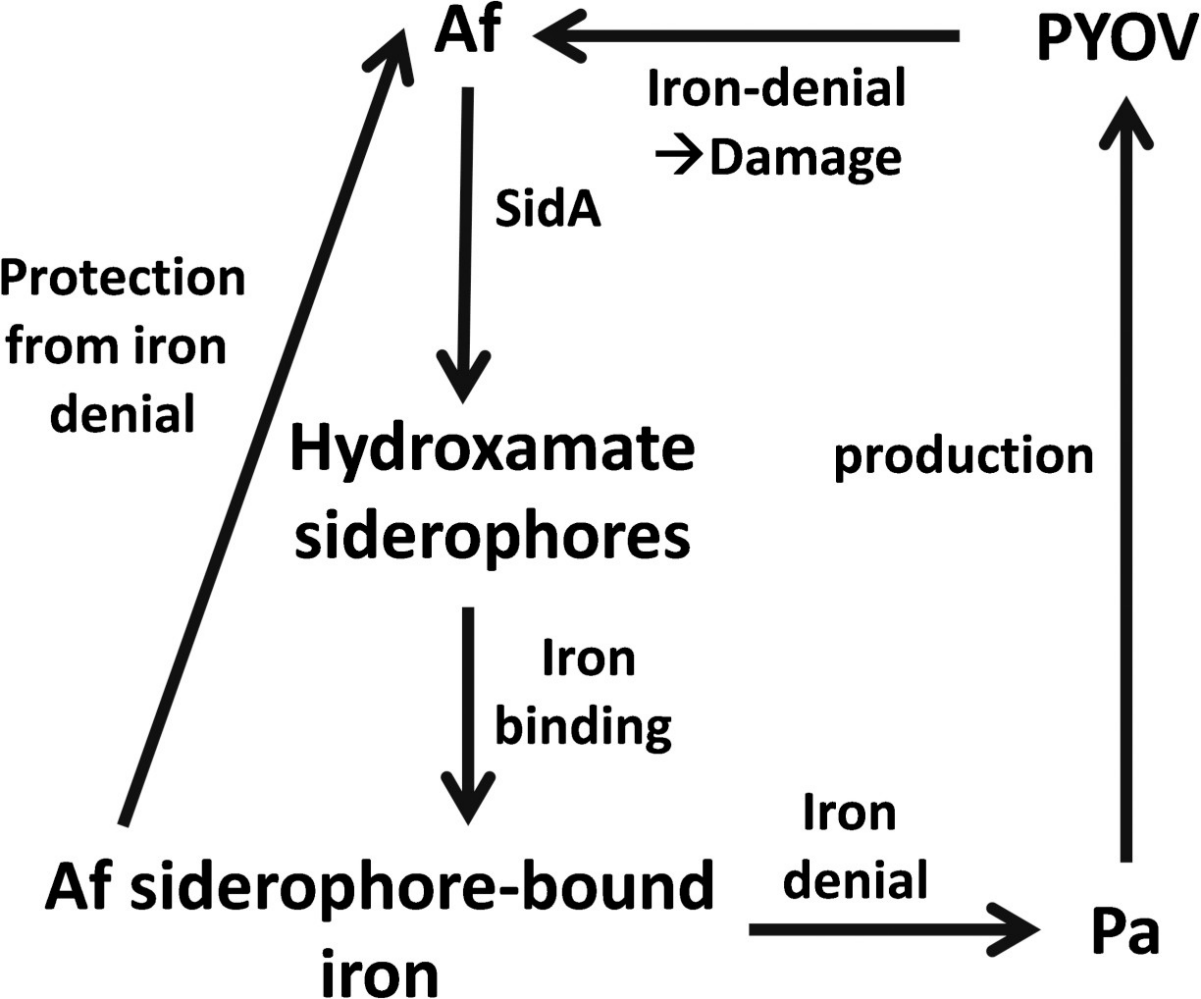
Summary. In need for iron *P*. *aeruginosa* (Pa) produces its siderophore pyoverdine (PYOV). PYOV-chelated iron is not available to *A*. *fumigatus* (Af), resulting in iron deficiency and damage to the fungus. Anti-fungal activity in part is counter-balanced by SidA-dependent *A*. *fumigatus* hydroxamate siderophores, providing iron to the fungus, further denying iron from *P*. *aeruginosa*.

As summarized in Fig 7, for *P*. *aeruginosa* as well as *A*. *fumigatus* a lack of iron is the signal to increase production of siderophores [27,28]. Siderophores specifically chelate ferric iron with a high affinity [57]. Siderophores are of different types, based on the way the iron is complexed: phenolate-, catecholate-, hydroxamate-, carboxylate-, or mixed type of siderophores have been described [58].

The *P*. *aeruginosa* siderophore pyoverdine is a composite (mixed) siderophore comprising a peptide chain and a chromophore [59]. Pyoverdines bind iron with very high affinity, are able to acquire iron from transferrin, and their production is absolutely needed in mouse pulmonary infections [60–62]. We have described pyoverdine to be the *Pseudomonas*-derived key inhibitor of *A*. *fumigatus* in their intermicrobial competition via iron sequestration under low iron conditions [26]. We note that the loss of pyoverdine did not prevent *P*. *aeruginosa* anti-fungal activity completely, however. Pyochelin, the second siderophore of *P*. *aeruginosa*, is produced by all *P*. *aeruginosa* isolates, but its affinity for iron is much lower compared to pyoverdine [63,64]. Pyoverdine does not act as a xenosiderophore for *A*. *fumigatus* [26], thus withholding iron from the fungus, and inducing anti-fungal effects [26]. *P*. *aeruginosa* does not seem to be able to use *A*. *fumigatus* siderophores either. Our results show that Afsups provoke increased pyoverdine production by *P*. *aeruginosa*, indicating that there is a paucity of iron in the medium. If *P*. *aeruginosa* could use iron bound to *A*. *fumigatus* siderophores, there would be an abundancy of iron available to the bacterium, and hence no increase in pyoverdine production. We here for the first time provide evidence that *A*. *fumigatus* is able to use iron bound to its hydroxamate siderophores as the main defense against *P*. *aeruginosa* competition for iron. These findings are summarized in Fig 7. Our results using *A*. *fumigatus* mutants defective in hydroxamate siderophore production also indicate that additional defense mechanisms might be in place, since supernatants derived from these mutants still partially protected from *P*. *aeruginosa* toxicity. Other microorganisms have developed defense mechanisms against *P*. *aeruginosa* not based on protective siderophore production. *Candida albicans* appears to defend itself against *P*. *aeruginosa* in part by down-regulating *P*. *aeruginosa* siderophore production [65].

Anti-bacterial *A*. *fumigatus* supernatant effects as a reason for protective effects against *P*. *aeruginosa* anti-fungal activity are highly unlikely. In the presence of Afsup, *P*. *aeruginosa* is able to even produce more pyoverdine, which requires functional bacterial metabolism. Also, Afsup protects from *P*. *aeruginosa* supernatants produced without Afsup being present, and Afsup, as well as pure *A*. *fumigatus* siderophores, protect from pure pyoverdine. Additionally, Afsups derived from a giotoxin mutant affected bacterial growth To the same degree as wildtype Afsups. The most plausible explanation for anti-bacterial effects of Afsups is depletion of essential factors in the medium, especially that of iron.

To overcome iron starvation, *A*. *fumigatus* produces its own siderophores [35]. *A*. *fumigatus* is able to produce four hydroxamate-containing siderophores: ferricrocin (FC) as well as hydroxyferricrocin (HFC) for intracellular iron trafficking, and fusarinine C (FsC) as well as its derivative triacetylfusarinine C (TAFC) for extracellular iron scavenging [36,66,67]. The first step in the biosynthesis of all four hydroxamate-containing siderophores is catalyzed by the enzyme L-ornithine N5-monooxygenase, termed SidA [30,67]. SidA catalyzes oxygen and NADPH-dependent hydroxylation of L-ornithine to N5-L-hydroxyornithine, a crucial step for the biosynthesis of hydroxamate-containing siderophores [35]. In a similar fashion to *P*. *aeruginosa* siderophores, *A*. *fumigatus* siderophores are essential for pathogenesis, as the AfΔ*sidA* strain is unable to establish invasive aspergillosis in a mouse model [35,56]. We show that in contrast to wildtype *A*. *fumigatus* supernatants, supernatants derived from AfΔ*sidA* were unable to protect *A*. *fumigatus* biofilms from detrimental effects of *P*. *aeruginosa* supernatants, or pyoverdine. This finding indicates the relevance of *A*. *fumigatus* siderophores for protection of *A*. *fumigatus* from *P*. *aeruginosa*-induced iron denial, and was supported by our finding that *A*. *fumigatus* siderophores (FC as well as TAFC) each could partially protect *A*. *fumigatus* from detrimental *P*. *aeruginosa* effects (Fig 4A and 4B). Pure preparations of the sideropore TAFC protected *A*. *fumigatus* from pyoverdine, even after heat treatment (Fig 4C, and S5 Fig). Protection by TAFC and its desferri form DF-TAFC was about equal, indicating that TAFC very efficiently binds free iron in medium, before pyoverdine can do the same. TAFC-bound iron does not seem to be transferable to pyoverdine, and exclusively is available to *A*. *fumigatus*.

*A*. *fumigatus* lacking hydroxamate siderophores, especially of the extracellular type, was more susceptible to pyoverdine (Fig 4B). The most pronounced detrimental effects of pyoverdine were observed when all four siderophores were missing.

A lack of siderophores, especially owing to a loss in SidA, renders the fungus more sensitive to iron denial by either pyoverdine (Fig 5B), or the clinically used iron chelator deferiprone (DFP, Fig 5B). Siderophore deficiency even sensitized the fungus to effects of amphotericin B (AmB, Fig 5C). While sensitization to DFP might be expected knowing that iron chelation by pyoverdine powerfully inhibits the fungus, sensitization to AmB is more surprising. It might be that a struggle for iron takes away energy from the fungus, and dampens intrinsic defense mechanisms, or that the membrane action of AmB [53] may adversely affect iron flux in the fungus.

As a pharmacological analog to *sidA* knockout we used celastrol treatment [54]. Celastrol, a pentacyclic triterpenoid that belongs to the family of quinone methides, exerts potent anti-cancer and anti-metastatic [68,69], anti-inflammatory [70,71], and antioxidant [72] activities. Recently celastrol was identified as a noncompetitive inhibitor of SidA production [54]. Inhibition of SidA production by celastrol is detrimental to *A*. *fumigatus* growth [54]. We observed inhibitory effects of celastrol on *A*. *fumigatus* metabolism as well (Fig 6). Since celastrol has numerous effects [68–71] it can’t be excluded that effects on *A*. *fumigatus* are not solely owed to inhibition of siderophores production.

SidA-deficiency or addition of celastrol to *A*. *fumigatus* wildtype cultures resulted not only in reduced fungal growth [54], and reduced *A*. *fumigatus* biofilm metabolism (Fig 6), but also in increased sensitivity towards the iron chelator DFP. DFP is clinically used to treat iron overload, as in thalassemia major [73], but also interferes with iron needs of bacteria [74], and *A*. *fumigatus* biofilms [51]. Given that celastrol does not have unwanted effects on the host it might be quite useful in supporting anti-fungal therapy.

Previous studies have focused on *P*. *aeruginosa* products and their inhibition of *A*. *fumigatus*, at high *P*. *aeruginosa* product concentrations. Such studies have not considered the possible response of *A*. *fumigatus* at the onset of *P*. *aeruginosa* competition. We here show that *A*. *fumigatus* uses its siderophores to counter-balance iron denial by *P*. *aeruginosa*. *In vivo*, the winning microbe in this competition might be the one which unleashes its products first, and in the greatest quantity. *A*. *fumigatus* siderophores seem to also strengthen the fungus against certain types of therapy. Therefore, interference with siderophore production might boost existing therapy against *A*. *fumigatus*.

## Supporting information

S1 Fig Overview of the experimental setup. Af: *Aspergillus fumigatus*; Afsup: planktonic *A*. *fumigatus* supernatant, Pa: *Pseudomonas*; Pasup: planktonic *P*. *aeruginosa* supernatant, PYOV: pyoverdine;
(TIF)

S2 Fig Gliotoxin content in Afsup is not likely to affect *P. aeruginosa*. *P*. *aeruginosa* cells (5 x 10^7^ /ml) were incubated with planktonic supernatants (25%) derived from AF5322 wildtype, AFgliΔP (gliotoxin mutant), or AFgliPR (reversion of the gliotoxin mutant) at 37°C for 24h. Bacterial growth (A600: A), and pyoverdine (PYOV; A405) were measured, and relative pyoverdine concentration (B) was calculated using the quotient A405/A600. Statistics by t-Test: PA14 supernatant, not containing Afsup (white bar) vs. PA14 supernatants containing Afsup. Two or three asterisks = p ≤ 0.01 or p ≤ 0.001, respectively.
(TIF)

S3 Fig Reduction of nutrients affects bacterial growth, but does not result in protection of *A. fumigatus* from *P. aeruginosa* toxicity. *P*. *aeruginosa* cells (5 x 10^7^ /ml) were incubated in RPMI 1640 medium containing 25% 10AFsup, or 25% sterile water, at 37°C for 24h. A: Bacterial growth (A600) was measured. Supernatants derived from A were tested for toxicity against *A*. *fumigatus* biofilm formation (XTT assay: B). Statistics by t-Test: A: PA14 supernatant prepared without Afsup or water addition (white bar) vs all other bars. B: RPMI (while bar) vs. all other bars. Other comparisons as indicated by the ends of the brackets. Two or three asterisks = p ≤ 0.01 or p ≤ 0.001, respectively.
(TIF)

S4 Fig Afsup induces pyoverdine and protects from *P. aeruginosa* anti-fungal activity. A: RPMI was inoculated with PA14 wildtype or the PA14 mutant PaΔ*pvdD* (5x10^7^ cells/ml), with (black bars) or without (white bars) the presence of 25% 10AFsup, and incubated at 37°C for 24h. Pyoverdine production was measured. B: Samples produced in A were used in a BCAM assay, and compared to metabolism of 10AF forming biofilm in the presence of RPMI or 25% 10AFsup, incubated without bacteria. Statistics: t-Test, as indicated by the ends of the brackets. Two or three asterisks = p ≤ 0.01 or p ≤ 0.001, respectively.
(TIF)

S5 Fig TAFC and DF-TAFC are stable to prolonged heat treatment. A 10AF BCAM assay was incubated with RPMI, TAFC [10 μM], DF-TAFC [10 μM], either fresh or heat treated (90°C for 30 min), and combined with pyoverdine (not heated) [PYOV, 10 μM]. Fungal metabolism was measured by XTT assay. Control (RPMI incubation without heat treatment) was regarded as 100%. Statistics: t-Test, comparison: PYOV without heat treatment vs. all other PYOV-containing bars. Other comparisons as indicated by the ends of the brackets. One, two or three asterisks = p ≤ 0.05, p ≤ 0.01 or p ≤ 0.001, respectively. Comparison of heat treatment of PYOV to unheated PYOV is also shown.
(TIF)

S1 Table Data sets used in this study. (PDF)

## Abbreviations

Af: *Aspergillus fumigatus*
Pa: *P*. *Aeruginosa*
PS: planktonic *P*. *aeruginosa* culture filtrate
PYOV: pyoverdine
BCAM: metabolic assay of *A*. *fumigatus* biofilm formation on agar
DFP: deferiprone
AmB: amphotericin B
FC: ferricrocin
HFC: hydroxy-ferricrocin
FsC: fusarinin C
TAFC: triacetylfusarinine C
DF-TAFC: desferri-triacetylfusarinine C
CAS: chrome azurol S
