# Supplementary figures and images for: Intermicrobial interaction: Aspergillus fumigatus siderophores protect against competition by Pseudomonas aeruginosa
Source: PLoS One. 2019 May 8;14(5):e0216085. doi: 10.1371/journal.pone.0216085 (PMC6505954; doi:10.1371/journal.pone.0216085)

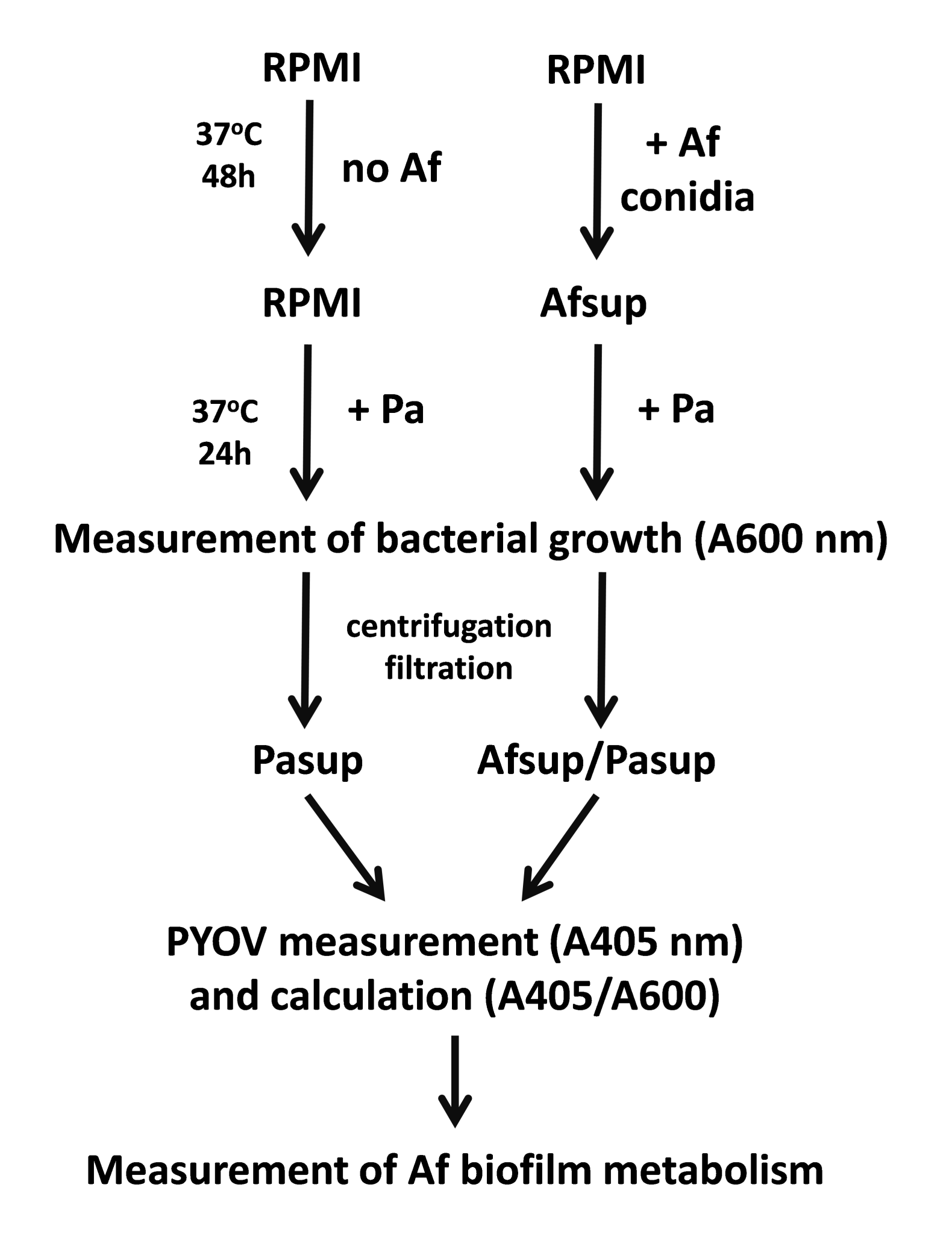

Supplement: S1 Fig — Af: Aspergillus fumigatus; Afsup: planktonic A. fumigatus supernatant, Pa: Pseudomonas; Pasup: planktonic P. aeruginosa supernatant, PYOV: pyoverdine; (TIF) [file pone.0216085.s001.tif]

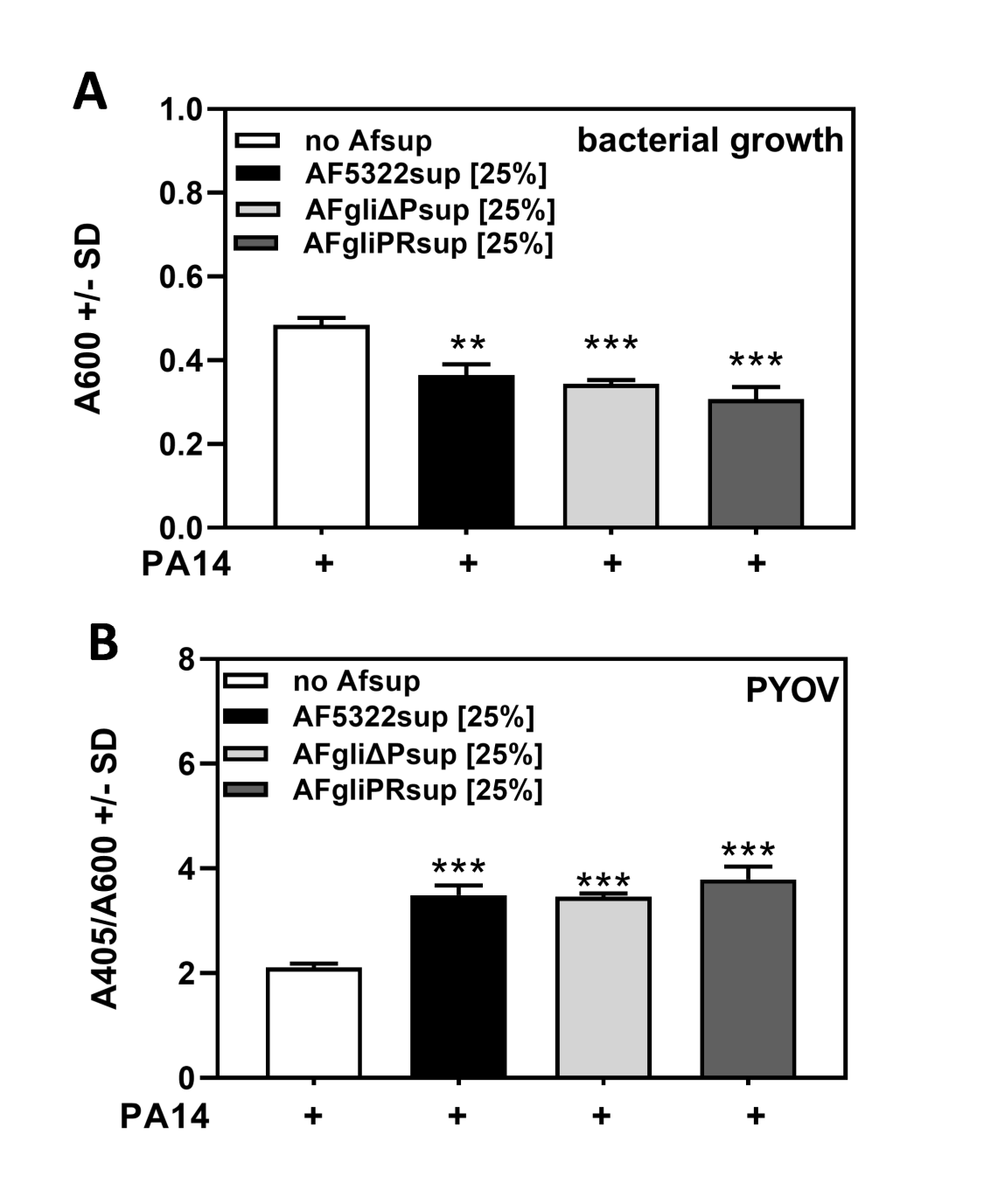

Supplement: S2 Fig — P. aeruginosa cells (5 x 10^7^ /ml) were incubated with planktonic supernatants (25%) derived from AF5322 wildtype, AFgliΔP (gliotoxin mutant), or AFgliPR (reversion of the gliotoxin mutant) at 37°C for 24h. Bacterial growth (A600: A), and pyoverdine (PYOV; A405) were measured, and relative pyoverdine concentration (B) was calculated using the quotient A405/A600. Statistics by t-Test: PA14 supernatant, not containing Afsup (white bar) vs. PA14 supernatants containing Afsup. Two or three asterisks = p ≤ 0.01 or p ≤ 0.001, respectively. (TIF) [file pone.0216085.s002.tif]

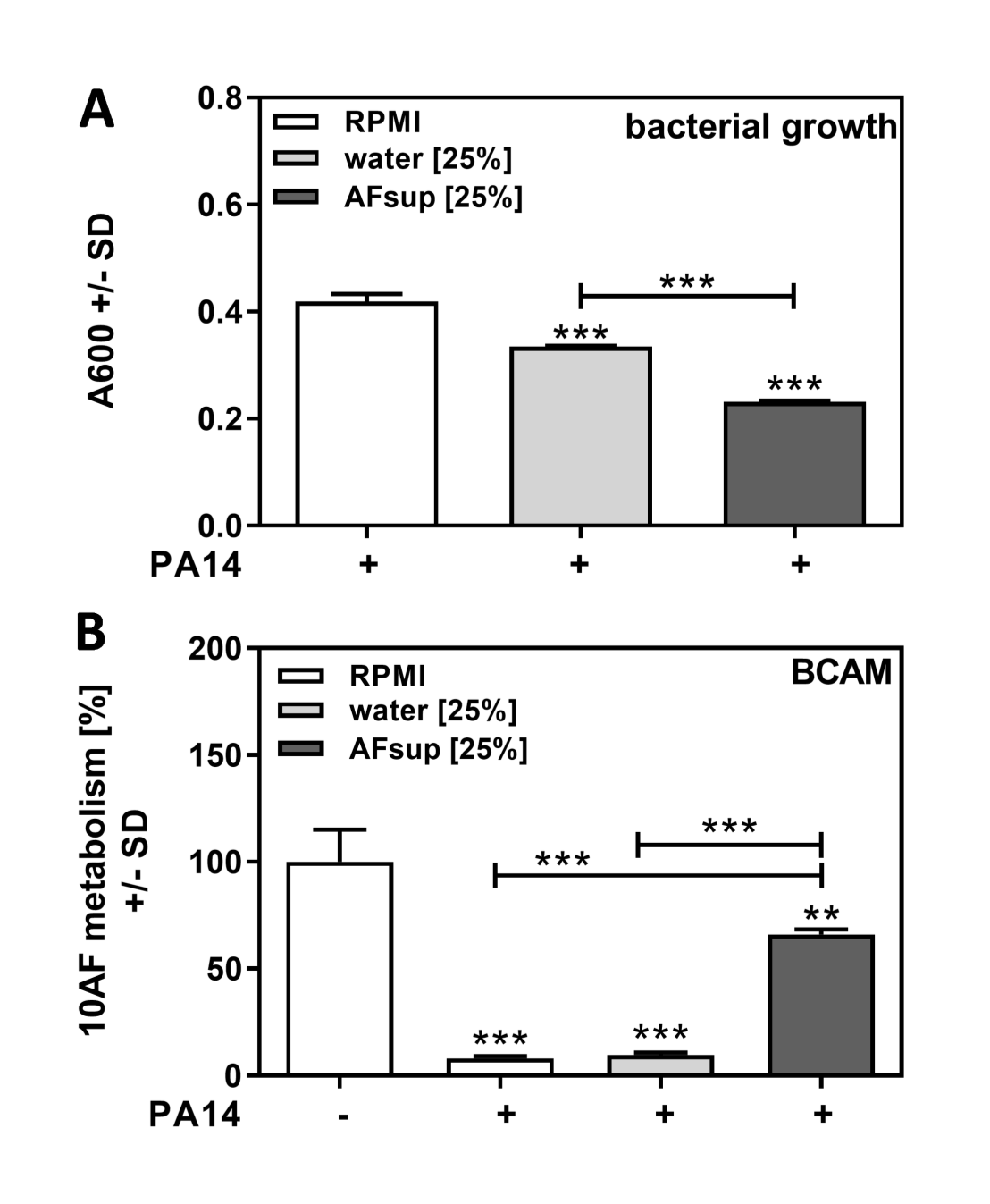

Supplement: S3 Fig — P. aeruginosa cells (5 x 10^7^ /ml) were incubated in RPMI 1640 medium containing 25% 10AFsup, or 25% sterile water, at 37°C for 24h. A: Bacterial growth (A600) was measured. Supernatants derived from A were tested for toxicity against A. fumigatus biofilm formation (XTT assay: B). Statistics by t-Test: A: PA14 supernatant prepared without Afsup or water addition (white bar) vs all other bars. B: RPMI (while bar) vs. all other bars. Other comparisons as indicated by the ends of the brackets. Two or three asterisks = p ≤ 0.01 or p ≤ 0.001, respectively. (TIF) [file pone.0216085.s003.tif]

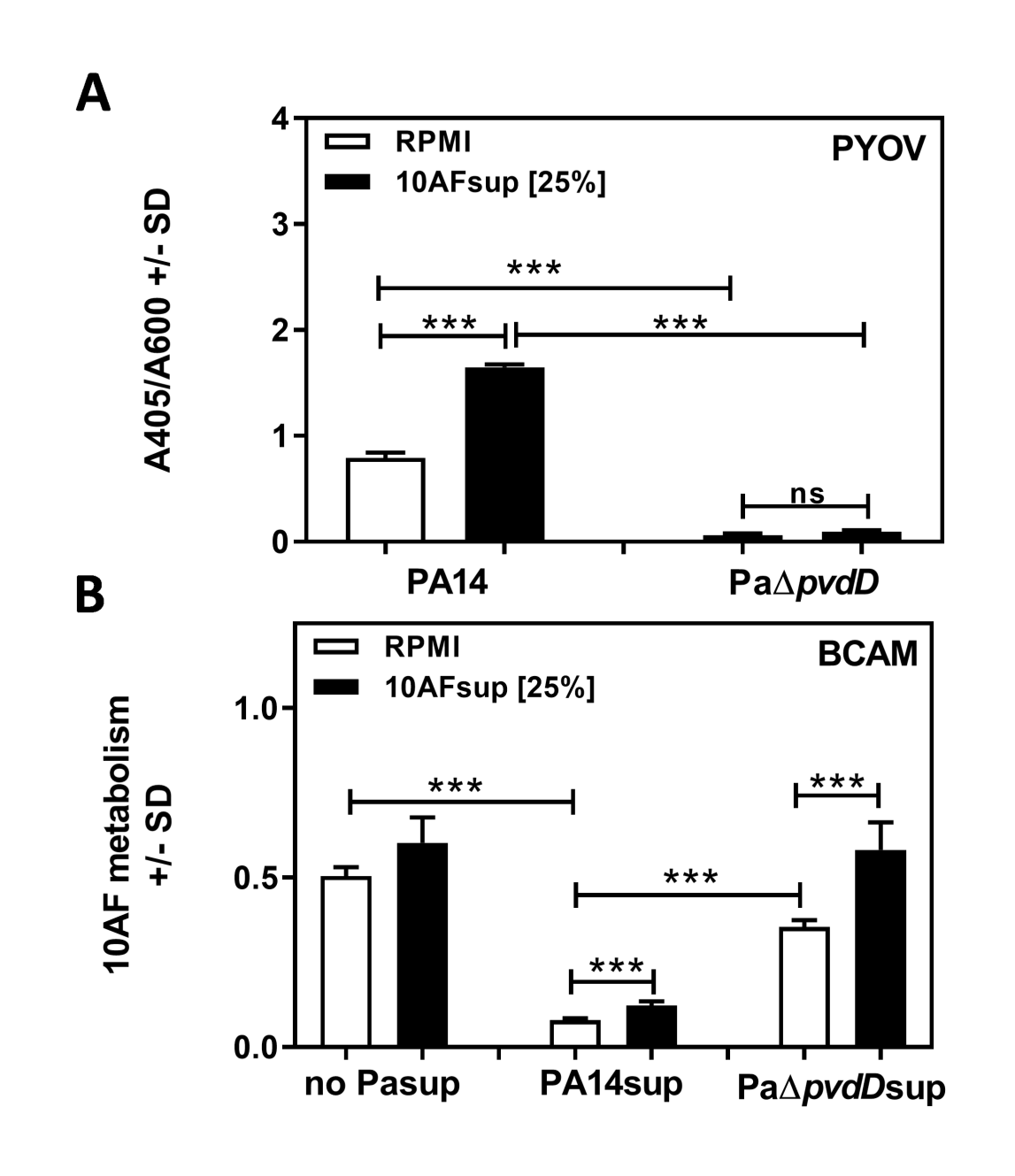

Supplement: S4 Fig — A: RPMI was inoculated with PA14 wildtype or the PA14 mutant PaΔpvdD (5x10^7^ cells/ml), with (black bars) or without (white bars) the presence of 25% 10AFsup, and incubated at 37°C for 24h. Pyoverdine production was measured. B: Samples produced in A were used in a BCAM assay, and compared to metabolism of 10AF forming biofilm in the presence of RPMI or 25% 10AFsup, incubated without bacteria. Statistics: t-Test, as indicated by the ends of the brackets. Two or three asterisks = p ≤ 0.01 or p ≤ 0.001, respectively. (TIF) [file pone.0216085.s004.tif]

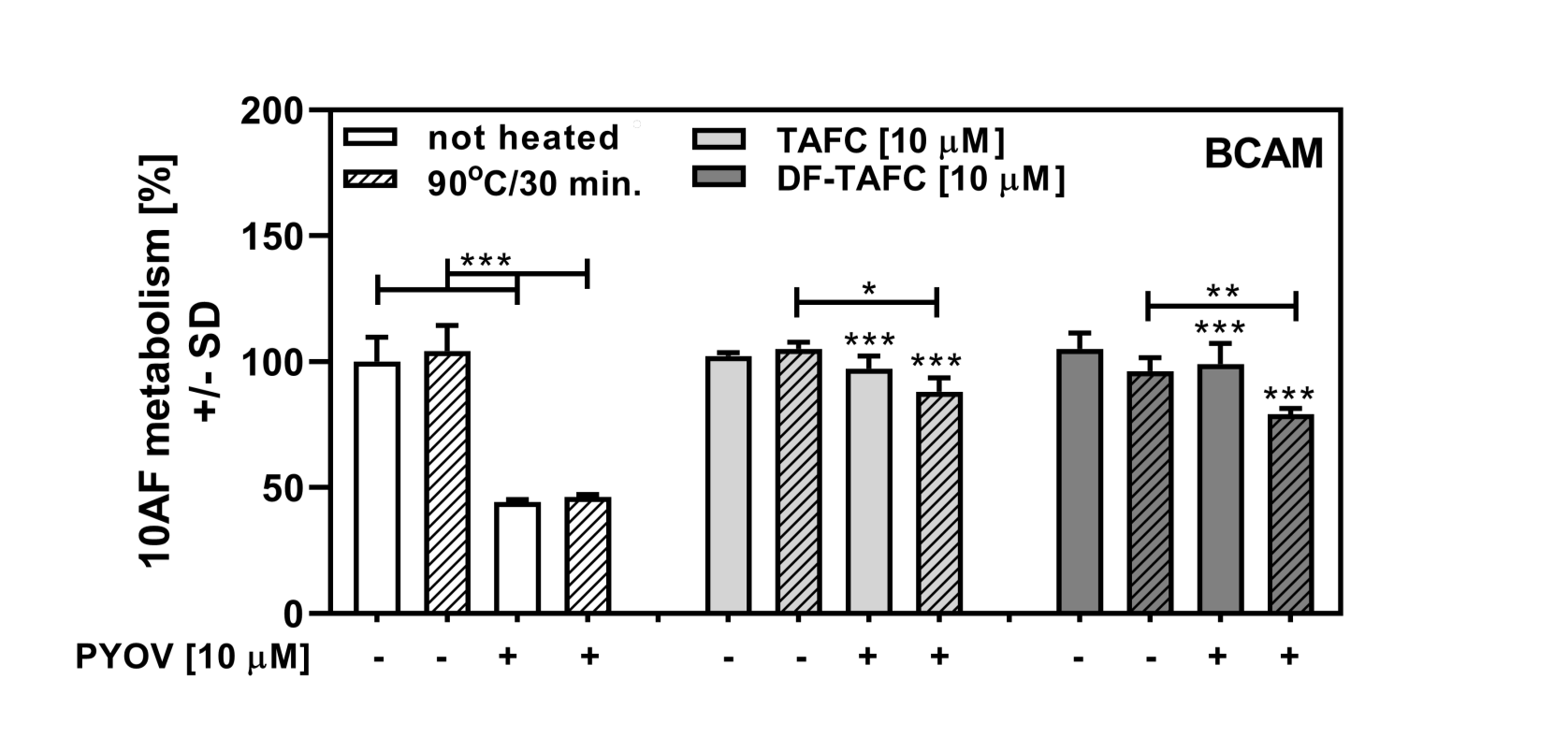

Supplement: S5 Fig — A 10AF BCAM assay was incubated with RPMI, TAFC [10 μM], DF-TAFC [10 μM], either fresh or heat treated (90°C for 30 min), and combined with pyoverdine (not heated) [PYOV, 10 μM]. Fungal metabolism was measured by XTT assay. Control (RPMI incubation without heat treatment) was regarded as 100%. Statistics: t-Test, comparison: PYOV without heat treatment vs. all other PYOV-containing bars. Other comparisons as indicated by the ends of the brackets. One, two or three asterisks = p ≤ 0.05, p ≤ 0.01 or p ≤ 0.001, respectively. Comparison of heat treatment of PYOV to unheated PYOV is also shown. (TIF) [file pone.0216085.s005.tif]
