# Supplementary material for: Intermicrobial interaction: Aspergillus fumigatus siderophores protect against competition by Pseudomonas aeruginosa
Source: PLoS One. 2019 May 8;14(5):e0216085. doi: 10.1371/journal.pone.0216085 (PMC6505954; doi:10.1371/journal.pone.0216085)
Supplement: S1 Table — (PDF) [file pone.0216085.s006.pdf]

Fig 1

	**RPMI/PA14**	**50% Afsup**	**40% Afsup**	**30% Afsup**	**20% Afsup**
**A**	0.79	1.92	1.64	1.45	1.22
	0.9	2.08	1.83	1.6	1.33
	0.88	2.08	1.83	1.57	1.35
	0.67				
	0.74				
	0.72				

	**RPMI/PA14**	**50% Afsup**	**40% Afsup**	**30% Afsup**	**20% Afsup**
**B**	0.551	0.479	0.515	0.525	0.545
	0.551	0.478	0.511	0.521	0.55
	0.565	0.474	0.501	0.537	0.544
	0.59				0.517
	0.59				0.513
	0.597				0.519

	**RPMI**	**PA14**	**Afsup 50/PA14**	**Afsup 25/PA14**	**Afsup 10/PA14**
**C**	109	11	57	60	32
	91	9	56	54	30
	97	7	55	53	33
	103	8	66	57	36
	101	12	88	69	56
	92	12	98	71	56
	110	12	94	67	48
	97	13	96	71	53

	**RPMI**	**PA14**	**Afsup 50/PA14**	**Afsup 25/PA14**	**Afsup 10/PA14**
**D**	100	71	91	90	85
	99	72	88	82	76
	97	72	83	86	81
	101	73	83	83	74
	102	71	95	87	81

Fig 1

**E**	**RPMI**	**Afsup25**	**Pasup25**	**Pasup10**	**A25Pa25**
	103	100	37	77	52
	96	108	37	71	52
	99	106	34	67	51
	93	102	36	67	50
	109	105	34	67	51
**F**	**RPMI**	**PYOV 5**	**PYOV10**	**AFsup25**	**P5A25**
	104	64	62	99	80
	98	69	61	113	83
	93	62	57	108	81
	101	70	56	104	85
	103	70	55	102	85
	107		45		
	96		48		
	105		49		
	85		50		
	108				
	112				
	109				
	81				
	93				
	104				
	112				
	97				
	102				
	88				

Fig 2

A	RPMI	Pasup 20%	Afsup 4C/Pa20	Afsup 56C 30 min/Pa20	Afsup 90C 30 min/Pa20
	100	46	78	76	80
	97	45	78	67	73
	106	46	74	62	74
	97	44	77	67	77

B	RPMI	PYOV 5	Afsup 3 months/P5	Afsup 6 months/P5	Afsup 9 months/P5
	89	50	73	71	67
	97	56	73	70	72
	113	53	67	65	59
	100	55	67	64	71

Fig 3

	AF13073sup/CAS	SidAsup/CAS
A	73	0.001
	72	0
	72	0
	72	0

	PA14/RPMI	PA14/AF13073sup	PA14/SidAsup	pvdD-/RPMI	pvdD-/AF13073sup
B	0.58	1.14	0.78	0.03	0.04
	0.62	1.19	0.84	0.06	0.07
	0.62	1.11	0.81	0.05	0.07
	0.73	1.51	1.08	0.05	0.06
	0.77	1.47	1.1	0.07	0.09
		1.43	1.13		0.08

	RPMI	AF13073sup	AfΔsidAsup	PA14/RPMI	PA14/AF13073sup
C	105	104	97	21	26
	95	101	102	18	24
	100	101	95	17	22
	100	99	99	16	20
	100	80	89	16	18
	97	97	83	16	19
	94	93	98	17	21
	106	102	99	14	21
	107	99	90	14	19
	97	106	86	14	20

	RPMI	10AFsup	AF13073sup	PYOV 5	PYOV 5/AF13073sup
D	105	125	133	25	114
	101	122	118	23	121
	91	122	127	23	130
	113	128	126	24	112
	91	135	129	23	102

Fig 4

	RPMI	RPMI/PYOV	AF13073	AF13073/PYOV	AfΔsidA
**A**					
	116	54	101	107	95
	109	48	94	92	90
	87	49	95	78	111
	88	47	109	82	103
	116	54	101	107	95

	RPMI AF13073	PYOV AF 13073	Pasup AF13073	RPMI AfΔsidA	PYOV AfΔsidA
**B**					
	114	88	42	104	22
	89	74	41	93	20
	97	74	47	101	19
	101	72	39	104	19
	99	80	41	98	19

	RPMI	PYOV 10	TAFC 10	TAFC 10 PYOV 10	TAFC 5
**C**					
	102	47	104	117	78
	96	47	94	83	98
	85	44	107	89	92
	117	49	117	91	101

Fig 5

B	RPMI AF13073	AF13073/ PYOV 10 uM	AF13073/ PYOV 5 uM	AF13073/ PYOV 2.5 uM	AF13073/ PYOV 1.25 uM
	99	24	49	87	101
	97	32	49	106	100
	97	32	49	92	98
	104	29	48	99	104
	99	27	51	89	100
	102	27	47	101	100

C	AF13073/RPMI	AF13073/DFP 2000 uM	AF13073/DFP 1000 uM	AF13073/DFP 500 uM	AF13073/DFP 250 uM
	101	18	59	90	94
	93	18	51	91	90
	108	25	58	79	87
	86	22	74	74	80
	114	18	57	71	97

D	AF13073/RPMI	AF13073/AmB 312 nM	AF13073/AmB 156 nM	AF13073/AmB 78 nM	AF13073/AmB 39 nM
	78	22	24	46	95
	112	22	27	44	88
	105	17	20	37	90
	110	17	20	39	76
	100	15	17	41	85

Fig 6

DFP 1000 uM/celastrol 5	DFP 1000 uM/celastrol 2.5	DFP 1000 uM/celastrol 1	DFP 500 uM/celastrol 5	DFP 500 uM/celastrol 2.5
18	37	34	36	60
17	30	39	53	69
18	25	36	42	59
18	28		46	59

Fig 1

A	10% Afsup	5% Afsup	2.5% Afsup	1% Afsup
	1.57	0.95	0.85	0.72
	1.18	1.03	0.96	0.8
	1.07	1.04	0.98	0.84
	1.08			
	1.15			
	1.15			

B	10% Afsup	5% Afsup	2.5% Afsup	1% Afsup
	0.604	0.584	0.6	0.594
	0.611	0.581	0.587	0.587
	0.575	0.571	0.586	0.582
	0.549			
	0.563			
	0.555			

C	Afsup 5/PA14	Afsup 2.5/PA14	Afsup 1/PA14
	19	21	18
	21	11	8
	22	10	7
	22	9	8
	39	27	21
	39	27	26
	38	30	25
	37	27	23

D	Afsup 5/PA14	Afsup 2.5/PA14	Afsup 1/PA14
	79	84	79
	78	79	77
	77	71	77
	74	72	73
	83	84	83

Fig 1

**E**	**A25Pa10**	
		122
		108
		103
		105
		119
**F**	**P10A25**	
		69
		68
		69
		68
		76
		67
		62
		64
		63

Fig 2

A **Afsup 3x thaw/Pas20**

	68
	62
	68
	68

B

	Afsup 12 months/P5	Afsup 12 months RT/P5
	70	67
	74	69
	67	61
	62	55

Fig 3

B

pvdD-/SidAsup
0.03
0.05
0.06
0.06
0.07
0.08

C

PA14/AfΔsidAsup	pvdD-/RPMI	pvdD-/AF13073sup	pvdD-/AfΔsidAsup
17	55	94	74
17	59	91	70
17	57	90	73
19	56	96	69
16	54	95	68
16	54	90	71
17	54	91	69
16	54	85	71
15	54	85	72
20	55	87	70

D

PYOV 5/AF13073sup
125
129
135
134
117

Fig 4

A

AfΔsidA/PYOV	AF46645	AF46645/PYOV	AfΔsidC	AfΔsidC/PYOV	AfΔsidF
62	105	106	90	94	110
66	100	111	98	105	99
59	100	108	103	102	93
56	95	107	109	105	98
62	105	106	90	94	110

B

Pasup AfΔsidA	AF46645/RPMI	AF46645/PYOV	AF46645/Pasup	RPMI AfΔsidC	PYOV AfΔsidC
21	100	57	33	124	48
21	97	56	33	94	54
21	107	54	33	97	49
21	103	54	29	91	47
21	102	53	31	94	48

C

TAFC 5 PYOV 10	DF-TAFC 10	DF-TAFC 10 PYOV 10	DF-TAFC 5	DF-TAFC 5 PYOV 10
81	113	99	102	102
86	90	90	106	116
82	85	100	105	91
78	102	96	89	96

Fig 5

B	AF13073/ PYOV 0.6 uM	AF13073/ PYOV 0.3 uM	AF13073/ PYOV 0.15 uM	RPMI SidA	AfΔsidA / PYOV 10 uM	AfΔsidA / PYOV 5 uM
	101	88	101	101	19	16
	98	95	95	94	16	15
	101	97	101	101	14	14
	100	113	104	108	14	14
	105	94	111	95	14	13
	93	93	104			

C	AF13073/DFP 125 uM	AfΔsidA /RPMI	AfΔsidA /DFP 2000 uM	AfΔsidA /DFP 1000 uM	AfΔsidA /DFP 500 uM	AfΔsidA /DFP 250 uM
	81	103	5	7	5	10
	97	85	3	7	5	10
	82	123	5	5	7	8
	93	90	3	11	8	10
	78	97	7			

D	AF13073/AmB 20 nM	AfΔsidA /RPMI	AfΔsidA /AmB 312 nM	AfΔsidA /AmB 156 nM	AfΔsidA /AmB 78 nM	AfΔsidA /AmB 39 nM
	110	94	15	29	31	63
	107	89	11	19	32	60
	115	100	15	15	34	73
	93	118	19	15	31	61
	98	97	16	13	29	61

Fig 6

DFP 500 uM/celastrol 1	DFP 250 uM/celastrol 5	DFP 250 uM/celastrol 2.5	DFP 250 uM/celastrol 1
70	55	78	87
71	56	88	86
73	58	69	78
	55	72	

Fig 4

A

AfΔsidF/PYOV	AfS77	AfS77/PYOV
82	107	103
77	97	98
76	105	99
75	92	100
82	107	103

B

Pasup SidC	RPMI AfΔsidF	PYOV AfΔsidF	Pasup AfΔsidF	RPMI AfS77	PYOV AfS77	Pasup AfS77
22	103	19	19	104	71	40
21	104	17	19	92	70	35
21	94	17	19	94	70	37
21	96	17	19	100	74	36
21	102	17	19	110	76	34

Fig 5

B	AfΔsidA / PYOV 2.5 uM	AfΔsidA / PYOV 1.25 uM	AfΔsidA / P 0.6 uM	AfΔsidA / P 0.3 uM	AfΔsidA / P 0.15 uM
	17	49	64	90	94
	17	52	74	81	93
	15	56	71	88	93
	14	52	85	87	87
	14	46	68	83	86

C	AfΔsidA /DFP 125 uM
	8
	8
	11
	8
	8

D	AfΔsidA /AmB 20 nM
	98
	95
	98
	85
	103
